# Gestational Diabetes: An Update 60 Years After O’Sullivan and Mahan

**DOI:** 10.1210/clinem/dgae709

**Published:** 2024-10-11

**Authors:** Harold David McIntyre, Ulla Kampmann, Tine Dalsgaard Clausen, Josephine Laurie, Ronald Ching Wan Ma

**Affiliations:** Mater Research, Faculty of Medicine, The University of Queensland, South Brisbane, QLD 4101, Australia; Steno Diabetes Center Aarhus, Aarhus University Hospital, 8200 Aarhus N, Denmark; Department of Clinical Medicine, Aarhus University, 8200 Aarhus N, Denmark; Department of Gynecology, Fertility and Obstetrics, Center for Pregnant Women with Diabetes, 2100 Copenhagen Ø, Denmark; Department of Clinical Medicine, University of Copenhagen, 2200 København N, Denmark; Mater Research, Faculty of Medicine, The University of Queensland, South Brisbane, QLD 4101, Australia; Department of Medicine and Therapeutics, The Chinese University of Hong Kong, Prince of Wales Hospital, Hong Kong; Hong Kong Institute of Diabetes and Obesity, The Chinese University of Hong Kong, Prince of Wales Hospital, Hong Kong; Laboratory for Molecular Epidemiology in Diabetes, Li Ka Shing Institute of Health Sciences, The Chinese University of Hong Kong, Hong Kong

**Keywords:** pregnancy, gestational diabetes, hyperglycemia, diagnosis, epidemiology, treatment

## Abstract

Sixty years after the landmark publication by O'Sullivan and Mahan, the current paper seeks to review and place into context the importance of gestational diabetes (GDM) both as a marker of increased risk of adverse maternal and fetal pregnancy outcomes and as an indicator of future maternal and infant health risks. We review the evolution of GDM as a diagnosis, the ongoing debate regarding diagnostic approaches, the underlying pathophysiology, and the epidemiology and the importance of GDM in the context of the global epidemics of diabetes and obesity. Focusing on clinical management, we review lifestyle-based therapy and pharmacotherapy for GDM, consider obstetric management, and outline the short-term and lifelong health risks posed by a GDM diagnosis.

The first case report of diabetes developing during pregnancy and (apparently) resolving postpartum dates to 1824 (1). The original case report and multiple subsequent studies identify excess fetal growth and adiposity as clear risks associated with hyperglycemia in pregnancy, and an association with other major pregnancy complications including hypertensive disorders of pregnancy, risk of birth trauma, neonatal hypoglycemia, and other short-term complications is well established. The term “gestational diabetes” (GDM) was first introduced by Carrington in 1957 (2) and gained prominence after the initial work O'Sullivan in 1961 (3), followed by the landmark publication by O'Sullivan and Mahan in 1964 (4). The current paper presents an overview of GDM in the 60 years since this paper. O'Sullivan and colleagues concentrated primarily on GDM as a risk marker for progression to overt diabetes. Current thinking acknowledges that a GDM diagnosis carries both short- and longer term health risks for both mother and baby (5).

At the time of these early studies in the Boston area, maternal obesity was uncommon—“16.2% (of women) were 20% or more over their ideal body weight” (4). In stark contrast, reports from the most recent NHANES survey in the United States report an obesity (body mass index > 30 kg/m^2^) prevalence of 39.9% in women aged 20 to 39 years (6) and a truly worrying prevalence of prediabetes/diabetes of 29.9% in girls aged 12 to 19 years (7).

Such demographic changes, in combination with the fact that glycemic testing is rarely undertaken before pregnancy, mean that many cases currently classified as “GDM” almost certainly have unrecognized prepregnancy hyperglycemia of varying degrees and likely represent a higher risk group in terms of pregnancy complications. Recommendations regarding early pregnancy detection of GDM vary. The American Diabetes Association (ADA) 2024 standards of care (8) recommend screening for early abnormal glucose metabolism using fasting venous plasma glucose of 110 to 125 mg/dL (6.1-6.9 mmol/L) or HbA1c of 5.9% to 6.4% (41-47 mmol/mol). The most recent guidelines from the American College of Obstetricians and Gynecologists (ACOG) recommend early testing aimed only at detecting overt diabetes and advise against early GDM screening (9). Recent studies such as the “Treatment of Booking GDM” trial (10) are starting to demonstrate differential risks between these groups, but definitive conclusions are not yet available and the majority of our current review will focus on “classical” GDM, generally detected between approximately 24 to 28 weeks’ gestation and defined as hyperglycemia, less severe than overt diabetes, which is first identified during pregnancy (11).

## GDM Definition: Personal vs Population Health

Despite multiple consensus conferences, the definition of GDM varies widely across the world. A summary of these divergent approaches is presented in Table 1. The International Association of Diabetes in Pregnancy Study Groups (IADPSG) hoped to achieve global consensus with the publication of its recommendations in 2010 (12), but this goal remains elusive. Because the HAPO study (13) demonstrated a continuous relationship between all oral glucose tolerance test (OGTT) glucose measures and adverse pregnancy outcomes, the selection of glucose thresholds for a dichotomous (Y/N) GDM diagnosis has been driven by exiting local protocols, fears of the practical and resource implications of “excess” GDM, and varying opinions regarding the level of hyperglycemia related risk that would justify a GDM diagnosis. The National Institutes of Health in the United States published a consensus report in 2013 (14) that concluded that there was insufficient evidence to change the traditional US-based approach to GDM using “2 step” (nonfasting glucose challenge followed by a formal fasting OGTT if the first step result is above threshold) using Carpenter—Coustan diagnostic thresholds (14). Subsequently, the IADPSG and traditional US approaches were compared in a large “pragmatic” randomized controlled trial (RCT) by Hillier et al (15) and a similar smaller study by Davis et al (16), both of which concluded that the IADPSG approach would at least double the frequency of GDM diagnoses without changing the population rates of adverse pregnancy outcomes. However, in both studies, the actual treatment received under each approach varied for <10% of the population, limiting the likelihood of any positive findings (17). The GEMS study from New Zealand (18) showed similar results at the population level, but also included a prespecified comparison of those women whose treatment actually differed depending on whether “tight” (IADPSG) or “less tight” (historical NZ) criteria were used to define GDM. In this subgroup, use of IADPSG criteria and subsequent active treatment resulted in a much lower frequency of severe adverse outcomes (0.5% vs 3.9%), preeclampsia (0.5% vs 5.6%). and large for gestational age (LGA) infants (6.2% vs 18.0%).

**Table 1.** Oral glucose tolerance test (OGTT) criteria for gestational diabetes

IADPSG 2010 glucose values at differing adjusted odds ratios vs mean*^a^* (12)	Sample size (n)	Fasting VPG mmol/L (mg/dL))	1-hour VPG nmol/L (mg/dL)	2-hour VPG mmol/L (mg/dL)	3-hour VPG mmol/L (mg/dL)
IADPSG OR 1.5	23 316	≥5.0 (90)	≥9.3 (167)	≥7.9 (142)	NA
IADPSG OR 1.75*^a^*	23 316	≥5.1 (92)	≥10.0 (180)	≥8.5 (153)	NA
IADPSG OR 2.0	23 316	≥5.3 (95)	≥10.6 (191)	≥9.0 (162)	NA
Glucose thresholds for a diagnosis of overt diabetes (IADPSG) or diabetes in pregnancy (WHO)*^b^* (12)	NA	≥7.0 (126)	NA	≥11.1 (200)	NA
**OGTT thresholds used for exclusion or inclusion in randomized trials**					
Crowther et al (19)	1000	≥7.0*^c^* (126)	NA	≥7.8*^d^* (140)	NA
Landon et al*^e^* (20)	958	≥5.3*^c^* (95)	≥10.0*^d^* (180)	≥8.6*^d^* (155)	≥7.8 (140)
**Selected alternative OGTT diagnostic thresholds for GDM**					
United Kingdom (NICE) (21)		≥5.6 (100)	NA	≥7.8 (140)	NA
India (DIPSI)—non fasting test (22)		NA	NA	≥7.8 (140)	NA
Denmark (DSOG 2023)		≥5.3 (95)	NA	≥9.0 (162)	NA
Carpenter–Coustan criteria (ACOG primary recommendation)*^f^* (23)	NA	≥5.3 (95)	≥10.0 (180)	≥8.6 (155)	≥7.8 (140)
NDDG criteria (ACOG alternative)*^f^* (23)	NA	≥5.8 (105)	≥10.6 (190	≥9.2 (165)	≥8.0 (145)

Abbreviations: ACOG, American College of Obstetricians and Gynecologists; DIPSI, Diabetes in Pregnancy Society of India; DSOG, Danish Society for Obstetrics and Gynecology; GDM, gestational diabetes mellitus; IADPSG, International Association of Diabetes and Pregnancy Study Groups; NA, not applicable; NDDG, National Diabetes Data Group; NICE, National Institute for Health and Care Excellence; OGTT, oral glucose tolerance test; VPG, venous plasma glucose.

^*a*^Thresholds are the mean glucose values during a 75-g OGTT at which the odds of birth weight, neonate percent body fat, and cord blood C-peptide level >90th percentile are 1.75 times the estimated odds of these outcomes at average glucose values. The alternative diagnostic thresholds corresponding to odds ratios of 1.5 and 2.0 compared to the mean are also shown. One elevated value is sufficient for a GDM diagnosis.

^*b*^Thresholds used to define overt diabetes or diabetes in pregnancy are the same as those used outside of pregnancy.

^*c*^Value for exclusion from trials.

^*d*^Value for inclusion in trials.

^*e*^Two or more elevated values at 1, 2, or 3 hours after glucose ingestion were required for exclusion or inclusion in the Landon trial.

^*f*^OGTT uses 100-g glucose load. Two or more elevated values are required for a GDM diagnosis.

Health economic analyses based directly on the landmark Landon (24) and Crowther (25) RCTs have reported satisfactory cost benefit parameters for GDM detection and treatment and cost modelling studies (26, 27) have reported benefits contingent on postpartum follow-up and preventive care for people with GDM.

Taken together, these trials have demonstrated that detection and treatment of “mild” GDM does not improve overall population level pregnancy outcomes but does reduce adverse outcomes for those who receive additional treatment because of a GDM diagnosis. These recent reports do offer additional insights, but so far have not resulted in major changes in clinical guidelines across the world.

## Pathophysiologic Mechanisms Underlying GDM

Normal pregnancy is characterized by increasing insulin resistance throughout pregnancy, which ensures adequate supply of nutritional substances to the fetus, including supply of glucose. Catalano et al showed that there is a uniform 50% to 60% decrease in insulin sensitivity with advancing gestation in both normal glucose-tolerant women and those diagnosed with GDM (28).

No single hormone has been found to completely account for the gestational insulin resistance, but placental hormones including human placental lactogen, prolactin, GH, estrogen and progesterone, and other hormones including cortisol and leptin are believed to play an important role (29). Kirwan et al (29) found no correlation between the placental reproductive hormones and cortisol and insulin sensitivity in late pregnancy but did report a significant correlation with TNF-α. Placental cytokines can play a role through secretion into the maternal circulation. McIntyre et al later reported that insulin growth factor binding protein 1, triglycerides, and leptin correlated significantly with maternal insulin sensitivity (30). As a new emerging area, extracellular vesicles derived from the placenta may modulate both physiological and pathological insulin resistance as inflammatory mediators such as cytokines are shown to be released in extracellular vesicles as part of fetomaternal communication (31).

Maternal obesity and adiposity also contribute to insulin resistance as an increase in maternal free fatty acids will exacerbate maternal insulin resistance. Accordingly, placental hormones, inflammation, and excess lipolysis can cause severe insulin resistance in liver, muscle, and adipose tissue in people with GDM (32). Other factors that are associated with reduced insulin sensitivity include advanced maternal age, a family history of type 2 diabetes (T2D), polycystic ovary syndrome, genetic factors, and physical inactivity (33).

The metabolic abnormalities underlying GDM, however, also include a defect in the insulin-secreting β cell. Increased insulin secretion is pivotal to the maintenance of normoglycemia in normal glucose-tolerant (NGT) pregnancies. Although GDM women generally show higher absolute insulin levels, they are insufficient to overcome the prevailing insulin resistance (34). Insulin secretion in response to IV glucose is also significantly enhanced in both NGT and GDM pregnancies, but most women with GDM display a reduced first-phase insulin response (34).

The defects in insulin resistance and insulin secretion most likely antedate pregnancy in many cases, especially in populations with high rates of diabetes and obesity (33).

## Epidemiology

It has been difficult to establish a clear picture of the global epidemiology of GDM and secular trends over recent decades. This is partly due to the lack of consensus on diagnostic criteria, which vary both within and between countries and regions (see Table 1), as mentioned previously. The lack of universal screening for GDM has also affected the validity of the estimated frequencies. Nevertheless, development of the IADPSG diagnostic criteria (12), adopted by the World Health Organization since 2013, helped to align diagnostic approaches across different regions, but has also resulted in increased GDM prevalence in most regions (35). A systematic review of 31 cohorts and cross-sectional studies with 136 705 women reported that implementation of the IADPSG criteria resulted in an overall 75% increase in the GDM prevalence compared to previous local criteria (relative risk, 1.75; 95% CI, 1.53-2.01) (36). Of note, there was significant heterogeneity in the changes in prevalence, highlighting potential ethnic differences in relation to fasting vs postprandial glucose concentrations during pregnancy (36, 37).

Despite these challenges, studies which have examined the prevalence of GDM using the same criteria have generally highlighted a marked increase in the incidence of GDM. For example, in data from Tianjin, China, prevalence of GDM increased by 3.5-fold between 1999 and 2010 through 2012, using consistent World Health Organization 1999 criteria (38), with further increase if the IADPSG criteria were applied. In a national cohort study from Denmark, again with unchanged criteria, GDM prevalence increased by 7% per year from 2013 to 2017, with substantial regional differences, highlighting potential influence from health care access as well as socioeconomic factors (39).

The increase in prevalence of GDM is partly due to increase in the prevalence of important risk factors for GDM, including increasing maternal obesity, as well as increasing maternal age. In a recent survey of global literature regarding GDM prevalence up to 2018 revealed that rates of GDM vary widely from around 1% to >20% (33, 40). GDM rates are in general lowest in Europe, and highest in the South East Asia region and the Middle-East/African region. In the HAPO study, the frequency of GDM, according to retrospectively applied IADPSG criteria, was 17.8% overall, but varied from 9.3% in Israel to 25.5% in Bellflower, California (41). In a recent pooled analysis of 57 studies for the IDF Diabetes Atlas, the pooled global standardized prevalence of GDM was 14.0% (95% CI, 13.97-14.04%) according to the IADPSG criteria, ranging from 7.1% in North America and the Caribbean, to 20.8% in South East Asia and 27.6% in Middle East and North Africa (42).

The risk factors for GDM have been well-established from different epidemiological studies and appear largely consistent, regardless of diagnostic criteria. These risk factors include increasing maternal age, maternal overweight or obesity, family history of diabetes, previous history of GDM, history of macrosomic baby, non-Caucasian ethnicity/race, and smoking (43). Furthermore, several nutritional factors have been identified through large-scale studies such as the Nurses Health Studies, which indicated the adverse impact of sugar-sweetened beverages, haem iron, animal fat, potatoes, and a high glycemic index (GI) diet. On the other hand, intake of dietary fiber, vegetable proteins, and physical activity, have been found to be protective against GDM (33). The close relationship between maternal diet and GDM has highlighted the importance of optimizing pre-conception and maternal diet (44) for the prevention of GDM.

## Pregnancy and Peripartum Issues in GDM

Gestational diabetes is associated with an increased risk of maternal and perinatal complications. The HAPO study demonstrated a linear correlation between oral glucose tolerance test values and complications, including preeclampsia, cesarean section, LGA, neonatal hypoglycemia, premature delivery, admission to neonatal care units (NICUs), shoulder dystocia, and hyperbilirubinemia (13).

### Maternal Pregnancy Risks in GDM

The most frequent GDM-related maternal complications and obstetric interventions during pregnancy and delivery are pregnancy induced hypertension, premature rupture of membranes, induction of labor, instrumental delivery, cesarean section, shoulder dystocia, and postpartum hemorrhage (45). These are attributed to the combined maternal insulin resistance and hyperglycemia, which leads to excess fetal growth and cardiometabolic stress in the mother (5).

A confounder adjusted large meta-analysis based on 156 observational studies of GDM vs NGT controls (including 35 studies of women with diet-treated GDM) reported increased odds of cesarean section (1.16; 1.03-1.32) in women with diet-treated GDM, whereas 63 studies comparing insulin-treated women with GDM with healthy controls found no differences in maternal outcomes. In the remaining 58 studies not reporting the use of insulin, women with GDM had increased odds of preeclampsia (1.46; 1.21-1.78), induction of labor (1.88; 1.16-3.04), cesarean section (1.38; 1.20-1.58), and premature rupture of membranes (1.13; 1.06-1.20). No clear differences were found in the odds of instrumental delivery, shoulder dystocia, or postpartum hemorrhage (45).

### Fetal and Early Neonatal Risks in GDM

Fetal/neonatal complications of GDM (46) are driven by maternal hyperglycemia resulting in fetal hyperglycemia and consequent fetal hyperinsulinemia and fetal anabolic effects. The end results are excess fetal growth (LGA and macrosomia), increased erythropoietin production leading to polycythemia and jaundice, neonatal hypoglycemia, respiratory distress syndrome, and need for NICU admission.

In the large meta-analysis mentioned previously, confounder-adjusted analyses showed an increased odds ratio of preterm delivery (1.51; 1.26-1.80), low 1-minute Apgar score (1.43; 1.01-2.03), macrosomia (≥4000 g) (1.70; 1.23-2.36), and LGA infants (1.57; 1.25-1.97) in pregnancies with diet-treated GDM compared to healthy controls, whereas in pregnancies with insulin-treated GDM there were higher odds of respiratory distress syndrome (1.57; 1.19-2.08), LGA (1.61; 1.09-2.37), neonatal jaundice (1.28; 1.02-1.62), and admission to NICU (2.29; 1.59-3.31) compared with healthy controls. Finally, in studies not reporting the use of insulin, GDM pregnancies had increased odds of congenital malformations (1.18; 1.10-1.26), preterm delivery (1.51; 1.19-1.93), macrosomia (1.48; 1.13-1.95), neonatal hypoglycemia (11.71;7.49-18.30), and admission to NICU (2.28; 1.26-4.13). No clear differences were found in the odds of stillbirth, neonatal death, low 5-minutes Apgar scores, low birth weight, and small for gestational age (SGA) infants (45).

Similar results regarding maternal and neonatal outcomes in pregnancies complicated by GDM have been reported in other observational studies (47, 48). A systematic review and meta-analyses of RCTs reported significant associations between GDM treatment and shoulder dystocia (OR, 0.40; 0.21-0.75), preeclampsia (2.5% vs 5.5%) and LGA (OR, 0.48; 0.38-0.62) (49). Furthermore, significantly lower gestational weight gain has been found in women treated for GDM compared to women with untreated GDM (18-20).

## Management of GDM: Diet and Physical Activity

The primary goal of treatment of GDM is to maintain normoglycemia and prevent excess fetal growth, adiposity, and other pregnancy complications (19, 50). The cornerstones of GDM treatment are a healthy diet and increased physical activity.

### Diet

Because carbohydrate (CHO) is the macronutrient that has the greatest impact on postprandial hyperglycemia, the focus of dietary modification is primarily on the type, amount, and distribution of carbohydrates (50). Most guidelines recommend daily CHO intake of 175 g per day and between 35% and 65% of dietary calories (51, 52) to promote appropriate fetal growth and cerebral development and to avoid ketonemia, which is reportedly associated with impaired cognitive and motor function in the offspring (33). CHO distribution is also important, and 3 main meals and 2 to 3 smaller snacks are recommended to avoid large CHO loads at each meal and reduce postprandial blood glucose peaks (53). GI and glycemic load (GL) are relevant in the management of GDM because diets rich in GI and GL may induce or exacerbate insulin resistance, whereas a low-GI diet is associated with improved glycemic control, reduced insulin requirements, lower cholesterol levels, and decreased inflammation (54).

In a systematic review and meta-analysis, including 9 RCTs, dietary interventions were evaluated in 884 women with GDM. The authors concluded that a diet with low GI reduced the use of insulin and improved offspring birth weight. Total energy restriction and low CHO diets did not change maternal or offspring outcomes (55).

The diurnal timing of CHO intake has also been investigated. A randomized crossover study examined the effects of a high-CHO morning intake vs a low-CHO morning-intake on glycemic variability and glucose control in 12 women with diet-treated GDM. A CHO distribution of 50% in the morning favored lower blood glucose and improvement in insulin sensitivity in women with GDM but resulted in higher glycemic variability (56). However, because the number of studies examining the impact of GI and GL on GDM are still limited and the findings are equivocal it is important that women with GDM receive an individualized nutrition plan developed in collaboration with an experienced dietician.

### Exercise

There is no consensus on the type, frequency, and duration of physical activity for women with GDM (50). Acute bouts of physical activity such as cycling for 20 minutes have been shown to reduce blood glucose excursions and insulin levels for 1 to 2 hours but have no demonstrable long-term effects on glycemia (57). Moderate intensity walking after a meal shows glucose lowering effects for 2 to 3 hours (58). Resistance exercise is associated with significantly lower 2-hour postprandial blood glucose levels and achieves better compliance compared to aerobic exercise in women with GDM (59, 60). To improve glucose profiles and reduce insulin resistance, moderate intensity physical activity for at least 30 minutes daily or 150 minutes weekly is recommended (61, 62).

### Weight Gain

Gestational weight gain (GWG) during pregnancy in women with GDM is the same as in women with NGT (50, 63). Limitation of GWG during dietary treatment has been shown to be associated with healthier fetal growth (64) and fewer pregnancy complications (65).

## Pharmacotherapy for GDM

Pharmacotherapy is indicated when self-monitored glucose levels during diet and exercise therapy are above the target range. Clearly, the former statement raises immediate issues regarding the (very limited) evidence underlying multiple local, national, and international guidelines regarding optimal/target glycemia in GDM. The most recent ADA (52) and American College of Obstetricians and Gynecologists (ACOG) guidelines (66) recommend the following targets: fasting glucose 3.9 to 5.3 mmol/L (70-95 mg/dL) and either 1 hour postmeal 6.1 to 7.8 mmol/L (110-140 mg/dL) OR 2 hours postmeal glucose 5.6 to 6.7 mmol/L (100-120 mg/dL). Continuous glucose monitoring offers a more detailed profile, especially for overnight glycemia, and may benefit some people with GDM, but there is currently insufficient evidence to support its routine use in GDM care (52).

The recent “TARGET” Study from New Zealand (18) compared tighter (fasting < 5.0/1 hour < 7.4/2 hour < 6.7 mmol/L; fasting < 90/1 hour < 133/2 hour < 120 mg/dL) glucose targets with less tight targets (fasting < 5.5/1 hour < 8.0/2 hour < 7.0 mmol/L; fasting < 99/2 hour < 126 mg/dL) using a step wedge design across 10 hospitals. TARGET reported a reduction (2.6%-1.3%) in the primary neonatal composite outcome of perinatal death, birth trauma, or shoulder dystocia with tighter glucose targets, but the secondary composite maternal outcome including major hemorrhage, coagulopathy, embolism, and obstetric complications was increased in the tighter target group (5.9% vs 3.0%).

One earlier, nonrandomized retrospective cohort study comparing 2 centers in Australia (67) also compared tighter (fasting < 5.0 mmol/L/2 hour < 6.7 mmol/L; fasting < 90/2 hour < 121 mg/dL) with less tight (fasting < 5.5 mmol/L/2 hour < 7.0 mmol/L; fasting < 99/2 hour < 126 mg/dL) treatment targets. This study reported no difference in the primary outcome of LGA infants, but reduced neonatal hypoglycemia, jaundice, and respiratory distress in the cohort treated to tighter targets at the expense of higher frequency of insulin used and increased obstetric interventions. Thus, evidence is mixed, and local consensus guidelines appear likely to predominate in the immediate future.

**Insulin therapy** remains the mainstay of pharmacotherapy for GDM. It is effective and the absence of any substantial transplacental transfer implies fetal safety. A variety of human insulin and insulin analogue preparations and regimens are used, depending on individual glycemic profiles, local availability, and cost considerations (33). Detailed consideration of their relative merits is beyond the scope of this review. The major problems relating to insulin therapy are the therapeutic burden for the pregnant woman (discomfort, fear of injections, and cost), the risk of hypoglycemia, and the increased health care resources required for insulin initiation and stabilization.

**Sulfonylurea therapy** (in particular, glyburide) became commonplace in GDM, especially in the United States, after the publication of the landmark RCT by Langer et al in 2000 (68), accompanied at the time by apparently reassuring data regarding absence of transplacental passage of glyburide (69). However, with the use of more sensitive assays, it has been clearly demonstrated that glyburide does cross the placental barrier (70), with widely variable concentrations seen in the fetal circulation. Larger scale pharmacovigilance studies have shown increased risks of macrosomia and neonatal hypoglycemia with glyburide therapy (71). These findings argue strongly against its routine use.

**Metformin therapy** remains the first-line option in T2D outside pregnancy and has been extensively studied in GDM (72-74). It has the advantage of being cheap and orally administered and the disadvantages of frequent gastrointestinal upset and the less common risk of vitamin B12 depletion and rarely lactic acidosis. Metformin is known to freely cross the placenta and most concerns regarding its use relate to potential “off-target” effects because of its widespread actions on cell maturation and differentiation (75). In published trials and meta-analyses, it appears safe and efficacious during pregnancy, likely with the benefit of reduced gestational weight gain (74, 76). In approximately 30% to 50% of women with GDM treated with metformin, supplemental insulin treatment is required to achieve glycemic goals (74). The recent EMERGE RCT from Ireland (77) showed no improvement in a primary composite outcome when metformin was routinely commenced in GDM women (regardless of glycemic levels on home testing), but did report lower rates of insulin initiation and reduced rates of LGA infants. The MOMPOD RCT (78), which recruited both women with T2D and early GDM, again showed no benefit with metformin treatment on a composite outcome, but also reported improved glycemic control and lower rates of LGA in the metformin-treated group. Metformin has been endorsed for first-line use in GDM in the United Kingdom (21) and considered an appropriate potential treatment in other countries (79), but these recommendations have been vigorously disputed (80). Beyond the theoretical concerns engendered by its pleiotropic pharmacologic effects, concerns have been raised by conflicting data regarding long-term effects on growth and adiposity of infants exposed to metformin in utero (81-83). Conclusions from animal models also remain inconclusive (84). As a result of these unresolved controversies, use of metformin in GDM is highly variable around the world, ranging from first-line therapy to total avoidance. Consensus on its use appears unlikely in the foreseeable future and local policies are likely to predominate.

**Other glucose-lowering agents** including dipeptidyl peptidase 4 inhibitors, sodium glucose cotransporter 2 inhibitors, alpha glucosidase inhibitors, meglitinides, thiazolidinediones, and glucagon-like peptide 1 agonists have minimal clinical data for efficacy and safety during pregnancy. Animal studies of early pregnancy exposure to glucagon-like peptide 1 agonists have shown fetal growth restriction and skeletal abnormalities (85), but human observational data appear reassuring (86). None of these agents can currently be recommended for the treatment of GDM.

## Obstetric Management

Evidence regarding obstetric management, monitoring, and timing of delivery in women with GDM is limited and recommendations are primarily based on consensus. A descriptive overview of National and International guidelines on diagnosis and management of GDM from 2021 included 7 guidelines (87): the National Institute for Health and Care Excellence in the United Kingdom, the International Federation of Gynecology and Obstetrics (11), the Australasian Diabetes in Pregnancy Society (88), the Society of Obstetricians and Gynecologists of Canada (SOGC_2019_) (89)), the American College of Obstetricians and Gynecologists (ACOG_2018_) (23), the ADA, and the Endocrine Society (ES_2013_) (90). Recent recommendations from the newly updated Danish Society of Obstetrics and Gynecology (DSOG_2024_) on GDM management (91)), as well as updated guidelines from NICE_2020_ (21) and ADA_2024_ (66) have been considered. Guidelines from ADIPS, ADA, and ES did not include recommendations regarding obstetric management.

### Antenatal Monitoring of the Mother

GWG guidelines uniformly follow the recommendations from the Institute of Medicine (92), whereas protocols for measurement of blood pressure and urinalysis vary from every 1 to 2 weeks (ACOG_2018_, FIGO_2015_) to 4 weeks in uncomplicated diet-treated pregnancies (DSOG_2024_).

### Antenatal Fetal Surveillance

Ultrasound scans to evaluate fetal growth, amniotic fluid, and placental function are generally recommended from 28 weeks and until delivery, but recommendations regarding frequency vary from every 2 to 4 weeks (FIGO_2015_), 4 weeks (NICE_2020_, SOGC_2019_), to 6 weeks in uncomplicated diet-treated pregnancies (DSOG_2024_). ACOG_2018_ recommends ultrasound scans from 32 weeks in poorly controlled and insulin treated cases. Monitoring fetal surveillance by cardiotocography (CTG) is generally recommended but protocols vary from routine weekly CTGs starting from 36 weeks (SOGC_2019_) or 38 weeks (NICE_2020_) to only on indication (DSOG_2024_) or in poorly controlled cases from 32 weeks (ACOG_2018_). In general, guidelines recommend intensified monitoring of the fetus in the presence of additional complications (eg, hypertension, polyhydramnios, LGA, SGA), but details are not specified.

### Timing and Mode of Delivery

Maternal hyperglycemia and fetal macrosomia (≥4000 g) are associated with increased risks of perinatal death as well as serious complications including shoulder dystocia, birth trauma, and cerebral palsy. However, no high-quality studies exist to guide the decision of timing of induction of labor or planned cesarean section, and recommendations remain largely empirical. FIGO_2015_ recommends cesarean section if estimated fetal size ≥4000 g and ACOG_2018_ if ≥4500 g. Dependent on obstetric history and maternal stature, DSOG_2024_ recommends induction of labor if estimated fetal size ≥4000 g and cesarean section if ≥4500 g. In uncomplicated diet-treated pregnancies induction of labor is recommended from: 39 to 40+6 weeks (ACOG_2018_), 40 weeks (SOGC_2019_), 40 to 41 weeks (FIGO_2015_), or before 41 weeks (NICE_2020_, DSOG_2024_). Recommendations in insulin-treated pregnancies range from: 39 weeks (SOGC_2019_), 39 to 39+6 (ACOG_2018_), and at 40 weeks (DSOG_2024_). Consensus exists that induction of labor before 37 weeks is seldom justified, but in case of complications (eg, poor glycemic control, hypertension, polyhydramnios, LGA, SGA), timing of delivery should be advanced.

### Intrapartum Maternal and Fetal Care

Hyperglycemia is associated with placental dysfunction. DSOG_2024_ recommends intermittent CTG intrapartum in diet-treated uncomplicated pregnancies and continuous CTG in insulin treated or otherwise complicated pregnancies. To reduce the risk of neonatal hypoglycemia, a maternal glucose target range of 4 to 7 mmol/L (72-127 mg/dL) is recommended during delivery (NICE_2020_, FIGO_2015,_ ES_2013,_ DSOG_2024_), monitored every 1 to 2 hours (NICE_2020_, DSOG_2024_).

### GDM: Psychological Factors and Stigmatization

The diagnosis of GDM may affect wellbeing and lead to social isolation and stigmatization (84-86). In a large review based on 44 studies, women with GDM reported both external stigma from health care personnel and relatives and internalized stigma, such as guilt and shame. Stigma can lead to avoidance of screening and evading dietary recommendations and blood glucose measurements. To avoid stigma, it must be kept in mind that the language is powerful and should be neutral, respectful, nonjudgmental, and based on strengths, facts, and actions that foster collaboration between patients and health care providers (93).

## Early Postnatal Care

Following birth, women diagnosed with GDM generally experience a reduction in glucose levels without pharmacotherapy. However, given the high prevalence of preexisting (undiagnosed) hyperglycemia before pregnancy, up to 1 in 3 women will have ongoing hyperglycemia consistent with diabetes or prediabetes (94, 95). Considering this and the longer term significant metabolic and cardiovascular risk conferred by GDM, early postnatal glucose tolerance testing (OGTT) or fasting glucose is recommended in most countries (21, 52). Unfortunately, many women fail to attend and reported follow-up testing rates vary from 23% to 58% (94, 96, 97). Reasons for nonattendance are likely complex and codesign of processes with consumers and ownership of the responsibility by clinicians is required to improve retesting rates (98).

Lawrence et al demonstrated that reminders in the form of phone, email or short message service increased the odds of a postnatal visit by 3 times compared with no reminders (99). An Indian study demonstrated that phone call reminders and a pragmatic approach to testing the woman at home (or her mother's home) resulted in a 95.8% follow-up rate (100). Careful consideration of women's health attitudes is also undoubtedly key to improving adherence. In hospital postpartum, glucose tolerance testing has also been reported to improve follow up (101) but is likely only feasible in health care systems in which inpatient postpartum care is routinely provided. In 2020, Finlayson's meta-synthesis of patient attitudes revealed that women valued assistance in supporting maternal self-esteem, competence, and autonomy postpartum. They recommended acknowledgment of the adaptation to changed intimate and family relationships and support for efforts to regain health and wellbeing for the dyad (102). Systems that are woman centered, use technologies that reduce barriers to access, and include clear messages from the health care providers will allow for better postnatal testing and early metabolic intervention in this high-risk group.

## Long-term Issues Following a GDM Pregnancy

### Maternal Health Following GDM

In addition to the short-term risk of pregnancy complications, long-term chronic disease risks are also increased in women with previous GDM. As previously mentioned, the defects in insulin resistance and insulin secretion found in women with GDM frequently antedate pregnancy, thus clearly the risk of developing T2D postpartum is substantial. Thus, a Finnish study showed recently that the relative risk of T2D was 11-fold for women with previous GDM compared to women without previous GDM (103). This is in line with a large meta-analysis by Vounzoulaki et al (104), who evaluated 20 studies including 1 332 373 individuals (67 956 women with GDM) and found that the women with a history of GDM appeared to have a nearly 10-fold higher risk of developing T2D compared to those with a normoglycemic pregnancy. Another study including 30 cohort studies with 2 626 905 pregnant women found that women with prior GDM had a 7.76 unadjusted pooled risk of diabetes compared with women without GDM, whereas the adjusted risk was 18-fold. Elevated body mass index, weight gain (105, 106), and a requirement for insulin treatment during the index GDM pregnancy (106) are associated with higher risks of progression. The risk of developing T2D after GDM was highest during the 3 to 6 years after GDM (107). Thus, the risk of T2D declines with time after pregnancy, but as shown in a large cohort study (108), the risk remains elevated even after more than 35 years following the diagnosis of GDM. Lifelong screening for T2D after GDM is therefore recommended.

In addition to the increased risk of T2D, women with previous GDM also have an increased risk of major cardiovascular disease (adjusted hazard ratio [HR], 1.69 [95% CI. 1.55-1.84]), hypertension (adjusted HR, 1.89 [95% CI, 1.82-1.96]), dyslipidemia (adjusted HR, 4.48 [95% CI, 4.28-4.69]), and venous thrombosis (adjusted HR, 1.32 [95% CI, 1.16-1.50]). Those women who were treated with insulin during pregnancy and who subsequently developed overt diabetes showed an exacerbation of the risk estimates (109). Moreover, the risk of chronic kidney disease is also significantly elevated after GDM, regardless of development of diabetes and hypertension (110).

Recent studies have addressed heterogeneity among women with GDM and suggest that future studies should focus on a deeper understanding of the genotypes and phenotypes related to the comorbidity and risk assessments of women with GDM to construct a more personalized follow-up program (111, 112).

### Offspring Health Following GDM

A growing and alarming body of evidence suggests that a hyperglycemic intrauterine environment may contribute to the rising pandemic of obesity and diabetes.

### Animal Studies

Animal (principally rodent) intervention studies have shown that intrauterine hyperglycemia is associated with anatomical changes in hypothalamic and hippocampal areas of the brain as well as increased risk of abnormal glucose tolerance, diabetes, obesity, insulin resistance, and cognitive disturbances in the offspring (113). Furthermore, the increased cardiometabolic risk can be inherited through several generations, driven by largely unknown pathways (114). Transplantation of pancreatic islet cells before the last part of pregnancy has been shown to prevent these effects (115, 116).

### Human Studies

Human RCTs in women with diabetes have been sparse but showed that reducing the level of maternal hyperglycemia prevents short-term adverse perinatal outcomes like neonatal adiposity. Human cohort studies consistently show that offspring exposed to intrauterine hyperglycemia (from GDM, T2D, or type 1 diabetes) are at increased risk of obesity, insulin resistance, impaired insulin secretion, T2D, and cardiovascular dysfunction compared to children from the background population. These adverse effects may be gender-specific and potentiated during the life course, driven by largely unknown pathophysiologic pathways, possibly including epigenetic mechanisms (117-123). The HAPO Follow-up Study, given that hyperglycemia was blinded and untreated during pregnancy, has in particular highlighted the association of maternal hyperglycemia with offspring adiposity, independent of maternal obesity (121, 122). Current GDM therapy has not been proven to attenuate the offspring risks of adiposity and cardiovascular risk factors in studies extending to age 5 to 10 years (124, 125). However, these substudies were not powered to examine long-term outcomes, and these observations are not definitive.

Studies of cognitive function, mood, and behavior in diabetes-exposed offspring are conflicting. Some find increased risk of anxiety, depression, attention disorders, autism, and cognitive impairment in diabetes exposed offspring, as well as indications of dose-response associations with levels of hyperglycemia (117, 126, 127), whereas others do not (128, 129). In line with animal studies, recent human studies including prepubertal offspring of mothers with GDM have demonstrated both functional and anatomical alterations in the hypothalamic, hippocampal and cortical areas of the brains of GDM offspring, including signs of central insulin resistance and decreased cortical excitability and neuroplasticity (130-134).

### Breastfeeding and Infant Health

One previous study found increased adiposity in breastfed offspring of mothers with diabetes (type 1 diabetes or GDM). This was exclusively associated with breastfeeding during the first neonatal week, but not with duration of breastfeeding (135). Infants who received larger volumes of “banked” breast milk from NGT mothers showed less adiposity. This study has never been replicated and its significance remains uncertain. In contrast, more recent studies have demonstrated that among offspring of women with GDM, higher intensity breastfeeding was associated with slower infant ponderal growth (136). Other studies have demonstrated that more intensive breast feeding reduces the risk of maternal T2D after GDM (137). Overall, breast feeding provides advantages for the long-term health of both mother and baby.

Thus, the adverse effects of intrauterine hyperglycemia are potentially reversible and optimizing treatment of maternal hyperglycemia during pregnancy may have crucial importance for health and disease in future generations and should be the focus for primary preventive strategies.

### Prevention of GDM

Given the adverse impacts of GDM on short- and long-term maternal and infant health, much effort has focused on the prevention of GDM. Several large-scale RCTs have been conducted to examine the feasibility of preventing GDM, mostly through dietary and lifestyle interventions, among at-risk women. These trials have mostly been negative, with few exceptions, suggesting that initiating intervention during pregnancy to prevent GDM may be “too little, too late” (138). For example, in the UPBEAT trial, behavioral intervention initiated from gestation 15 weeks onwards in women with obesity did not reduce incidence of GDM or LGA infants (139). Nevertheless, in a systematic review of 19 RCTs on lifestyle intervention to prevent GDM, either diet or physical activity intervention was associated with an 18% (95% CI, 5-30; *P* = .0091) reduction in the risk of GDM in the pooled analysis, with the subgroup analysis suggesting that intervention was effective among women who received the intervention before the 15th gestational week, but less effective among those who received the intervention later in pregnancy (140). Recent advances in mobile health technology and smartphones have also elicited much interest in the potential for applications to reduce GDM. A systematic review of 16 RCTs with 7351 participants suggested that mHealth-based lifestyle interventions had a favorable impact on the prevention of GDM in pregnant women with overweight and obesity and warrants further investigations in larger RCTs (141).

Given that GDM is associated with 7- to 10-fold risk of subsequent progression to T2D (104, 107), and that offspring of mothers with GDM are also at increased risk of obesity and metabolic disorders (122), the focus has increasingly shifted to the prevention of DM post-GDM, and empowering mothers for life course prevention of DM (142, 143). This is particularly important given the burden of adverse outcomes in the long term are often mediated through progression to diabetes (144). In the Lancet Commissioned Report on Diabetes, it was recognized that prevention of future GDM and diabetes among women with GDM is one of the “low-hanging fruits” in terms of diabetes prevention (143). Likewise, the International Federation of Gynecology and Obstetrics (FIGO), recognizing the importance of pregnancy as a window of opportunity for life course prevention of noncommunicable diseases such as diabetes, has highlighted the importance of postnatal metabolic screening post-GDM, and engaging women in diabetes prevention (142). Several studies have highlighted the feasibility of engaging mothers early post-GDM (145-147), though with variable effects on short-term adiposity measures or metabolic outcomes. A systematic review also suggested that lifestyle intervention initiated within 3 years postpartum may be more effective in preventing postpartum diabetes compared to trials when the intervention was initiated later (148). Follow up from the Diabetes Prevention Program has also suggested that metformin may be particularly efficacious in reducing progression toward overt diabetes in women with a previous history of GDM (149, 150).

Given the potential intergenerational effects of maternal hyperglycemia, and the long-term impact on metabolic health in the offspring, it is important to recognize that targeting GDM and adiposity at different stages of the life course represent unique opportunities to break this intergenerational cycle of obesity and diabetes (143, 151-154).

## Summary and Future Directions

Despite the ongoing lack of consensus regarding specific diagnostic criteria, it is clear that GDM represents a major lifetime health challenge for women and their children because of its association with both immediate pregnancy complications and long-term health. Current evidence clearly demonstrates that pregnancy complications can be prevented by appropriate management during pregnancy, but widely differing diagnostic approaches and antenatal health care policies mean that the number of women who receive any form of GDM screening/testing and the number actually diagnosed with and treated for GDM varies greatly across the world. Perceptions of the “value” of GDM treatment and “appropriate” diagnostic thresholds appear driven largely by local consensus and concerns regarding the resources required for GDM treatment, rather than by published data.

Other ongoing areas of controversy, which require further well-designed and executed studies to provide a clear evidence base for clinical practice include (1) optimal glycemic targets to guide lifestyle interventions and progression to pharmacologic management; (2) the role of oral hypoglycemic agents and newer injectable agents in GDM management; (3) development and implementation of optimal strategies for monitoring of fetal growth and well-being in GDM pregnancies; and (4) the relative importance and therapeutic impact of earlier GDM diagnosis and treatment.

A GDM diagnosis also offers the possibility of identifying women at increased risk of cardiometabolic problems after pregnancy. Unfortunately, this challenge is rarely met with effective strategies for lifetime health improvement in women with previous GDM. Infants of women with GDM also carry lifetime health risks, but these have not been shown to be improved by current diagnosis and treatment of GDM.

**Figure 1.**
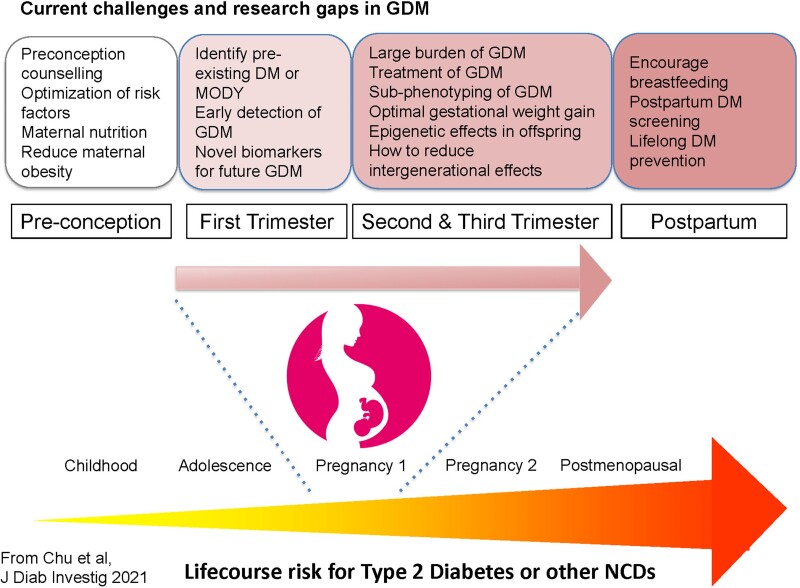
Life course view of the impact of gestational diabetes mellitus (GDM) on overall risk of lifetime risk for development of type 2 diabetes and other noncommunicable diseases (NCD). Note multiple potential timepoints for identification of risk and intervention.

In the current global context of increasing obesity, later childbearing, and a rising burden of chronic noncommunicable diseases (155), appropriate GDM detection and treatment provides an opportunity for improved lifetime metabolic health. A summary of GDM in a lifetime context is presented in Fig. 1. Reproduced with permission from *Journal of Diabetes Investigation*: 2021. Further research should be devoted to appropriately detecting GDM, to ensuring that treatment during pregnancy is effectively delivered without exacerbating maternal stress or imposing a burden of stigmatization and to the development and implementation of pragmatic, large scale programs to improve long term maternal follow up and management.

## Data Availability

Data sharing is not applicable to this article as no datasets were generated or analyzed during the current study.

## Abbreviations

ACOG: American College of Obstetricians and Gynecologists
ADA: American Diabetes Association
CHO: carbohydrate
CTG: cardiotocography
GDM: gestational diabetes mellitus
GI: glycemic index
GL: glycemic load
GWG
HR: hazard ratio
IADPSG: International Association of Diabetes in Pregnancy Study Groups
LGA: large for gestational age
NGT: normal glucose-tolerant
NICU: neonatal intensive care unit
OGTT: oral glucose tolerance test
RCT: randomized controlled trial
SGA: small for gestational age
T2D: type 2 diabetes
